# A Rare Case of Peritoneal Dialysis-Associated Peritonitis Caused by Neisseria sicca

**DOI:** 10.7759/cureus.59358

**Published:** 2024-04-30

**Authors:** Pushyami Satya Bandi, Ajit Brar, Rami Al-Handola, Yasaman Navari, Danielle Osterholzer

**Affiliations:** 1 Internal Medicine/Pediatrics, Hurley Medical Center/Michigan State University, Flint, USA; 2 Internal Medicine, Hurley Medical Center/Michigan State University, Flint, USA; 3 Infectious Disease, Hurley Medical Center/Michigan State University, Flint, USA

**Keywords:** infectious disease, esrd, neisseria sicca, peritonitis, peritoneal dialysis

## Abstract

Peritonitis is a critical complication in peritoneal dialysis, often leading to peritoneal dialysis (PD) treatment failure. We present a rare case of *Neisseria sicca* (NS)-related peritonitis in a 47-year-old male with multiple comorbidities. Despite NS's infrequent association with infections, prompt diagnosis and antibiotic therapy based on International Society for Peritoneal Dialysis (ISPD) guidelines led to a favorable outcome. This case emphasizes identifying uncommon pathogens in immunocompromised individuals and highlights the importance of prompt treatment to prevent morbidity.

## Introduction

Peritonitis is a significant, potentially life-threatening complication seen in patients receiving peritoneal dialysis (PD). Peritonitis related to peritoneal dialysis is a serious issue that can lead to treatment failure, which in turn may necessitate the removal of the catheter, discontinuation of PD, and a shift to hemodialysis. Peritoneal dialysis improves the quality of life in patients with end-stage renal disease (ESRD) by decelerating the decline in kidney function as well as providing the convenience of dialysis at home. Peritoneal dialysis-associated peritonitis (PDAP) is the most common source of infection in those patients. PDAP is associated with increased morbidity. Mortality rates increase in patients on peritoneal dialysis for more extended periods of time [1,2].

PDAP is diagnosed based on the International Society for Peritoneal Dialysis (ISPD) guidelines by the presence of two out of the following three criteria; presence of clinical features consistent with peritonitis such as abdominal pain and/or cloudy dialysis effluent, effluent with white blood cell count greater than 100 per microliter or > 0.1 × 10^9^ per liter with greater than 50% polymorphonuclear (PMN) leukocytes, and a positive dialysis effluent culture [3].

Neisseria sicca (NS) is a typically non-pathogenic gram-negative bacteria commensally present in the human oropharynx [4]. The Neisseria genus encompasses 25 species most commonly found on mucosal or dental surfaces in mammals. Historically, Neisseria sicca was rarely associated with any human infections but has been infrequently described as an atypical cause of endocarditis and meningitis in the literature. We present an exceedingly rare case of peritoneal dialysis-associated peritonitis secondary to N. sicca.

## Case presentation

A 47-year-old male with a known past medical history of end-stage renal disease (ESRD), on peritoneal dialysis (PD) awaiting a kidney transplant, coronary artery disease status post coronary artery bypass graft surgery, insulin-dependent diabetes mellitus, hypertension, and anemia secondary to ESRD presented to the emergency department after he was referred from his dialysis clinic for evaluation of abdominal pain associated with vomiting for three days before admission. The patient characterized the pain as crampy, 10/10 in intensity, and endorsed aggravation with movement and relief with rest. The patient undergoes peritoneal dialysis treatments at home through the peritoneal catheter. The patient reports that he recently attached the dialysis fluid bag for exchange and a few weeks before his symptoms appeared, there was technique failure with PD fluid leak from the cap. There was no history of chest pain, fever, shortness of breath, headache, or blurred vision.

Upon arrival at the emergency department, the patient was in severe pain, afebrile, hemodynamically stable with a blood pressure of 101/81 mmHg, heart rate of 87 beats per minute, and saturating well on room air. Laboratory investigations revealed a white blood cell (WBC) count within normal limits and mild elevation in neutrophils, elevated blood urea nitrogen (BUN), creatinine, phosphate, hemoglobin A1c, anemia, and hyponatremia as in Table 1.

**Table 1:** Initial laboratory values of the patient compared to reference ranges.

	VALUES	REFERENCE RANGE
White Cell Count	8.7	4.8-10.8 K/UL
Neutrophils	88	36-75%
Sodium	129	136-145 mEq/L
Blood Urea Nitrogen (BUN)	35	7-20 mg/dl
Creatinine	6.4	0.7-1.2 mg/dl
Phosphate	4.7	2.5-4.5 mg/dl
Haemoglobin	12.3	14-18 g/dl
Haemoglobin A1c	7.7	5.7-6.4%

**Figure 1:**
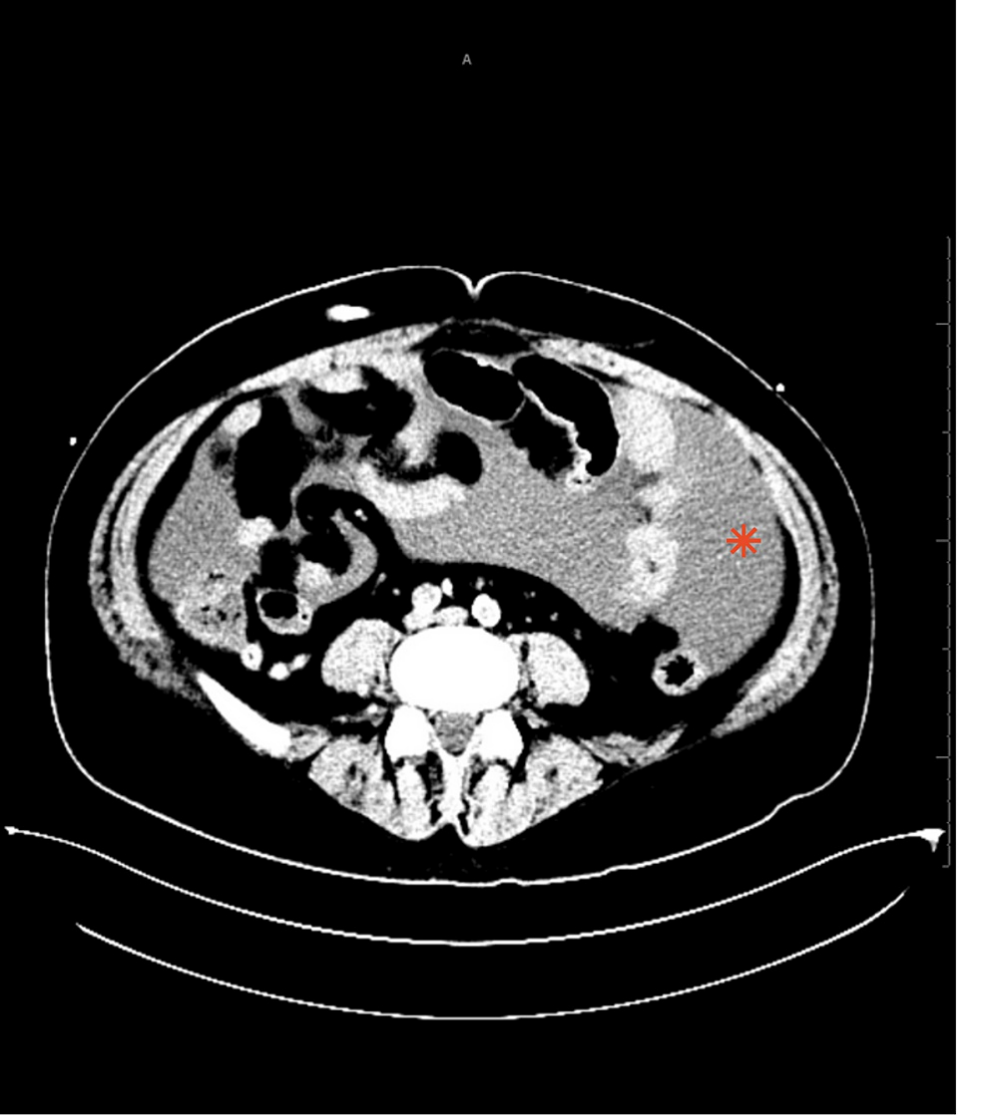
CT abdomen axial view showing the peritoneal fluid collection - Indicated by asterisk

On examination, the patient's abdomen was distended on palpation, and there was generalized abdominal tenderness without guarding or rebound tenderness with a positive fluid wave and shifting dullness. Computed tomography (CT) of the abdomen and pelvis was remarkable for peritoneal fluid as shown in Figure 1.

**Figure 2:**
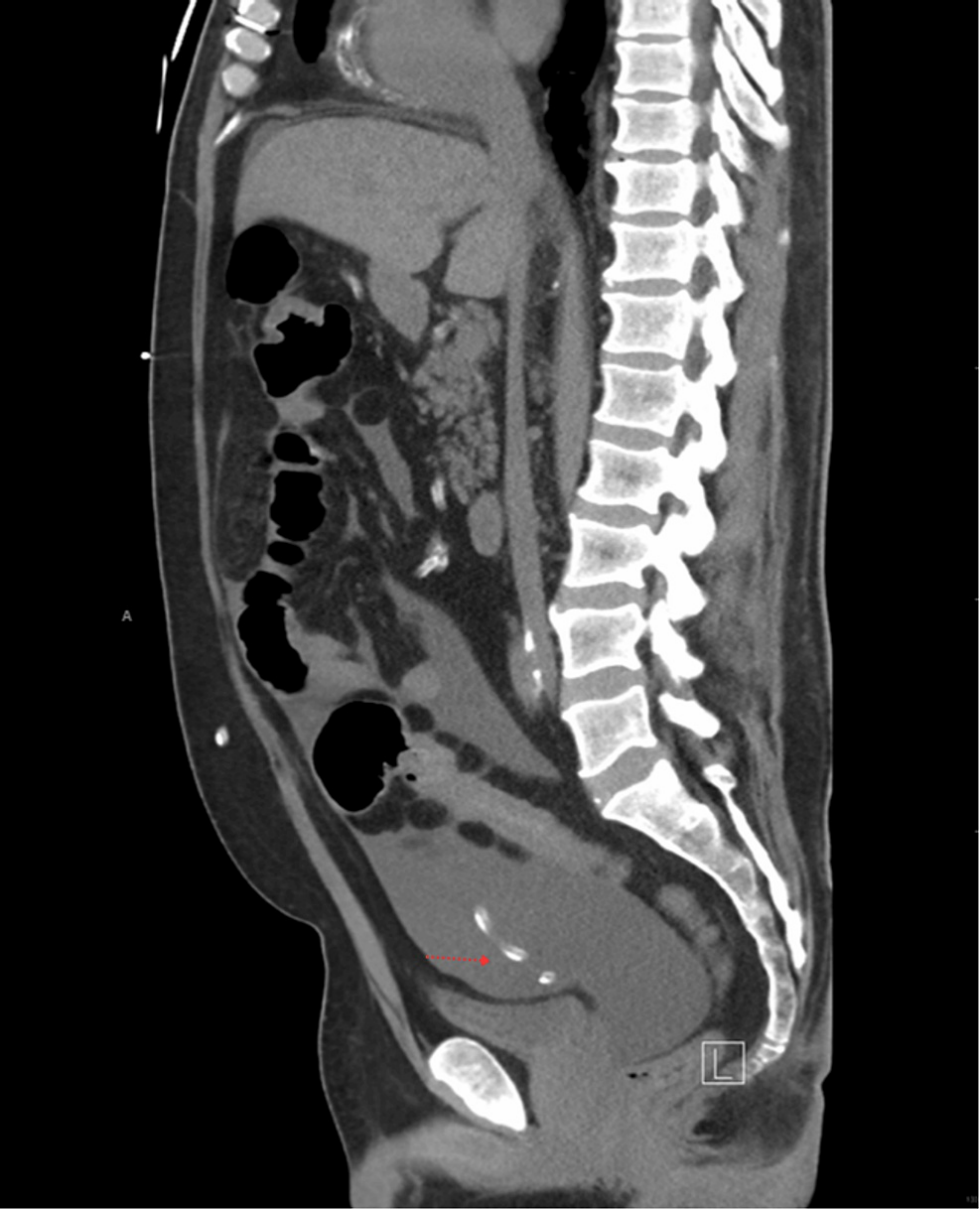
Sagittal view of CT abdomen and pelvis showing peritoneal fluid and peritoneal catheter.

Figure 2 shows the sagittal view of CT with the peritoneal fluid and peritoneal catheter of the patient.

Specimens of blood from peripheral veins and dialysis effluent were collected for testing. Neutrophilic leukocytosis [White blood cell (WBC) count 3000 with 60% neutrophils] on ascitic fluid analysis pointed towards the provisional diagnosis of peritoneal dialysis-associated peritonitis with an unknown organism. The patient was started on empiric intravenous antibiotics, cefepime, as well as vancomycin for broad coverage of bacterial peritonitis and was admitted to the medical floors for further management.

The patient underwent correction of electrolyte imbalance and continued to receive peritoneal dialysis as per protocol. After a detailed history and fluid analysis, PD-associated peritonitis was diagnosed, and the patient was shifted to intraperitoneal antibiotic vancomycin and cefepime from intravenous based on guidelines for PD-associated peritonitis. The patient was symptomatically managed for pain. Subsequent daily dialysis effluent counts initially showed rising white blood cell counts, with neutrophils accounting for 93% of cells. The cell count gradually fell over the next few days, along with reported pain and abdominal tenderness improvement. The patient’s dialysis effluent culture grew Neisseria sicca, and the blood cultures were negative. The patient responded well to antibiotics and dialysis effluent analysis showed a remarkable decrease in WBC cell count. Based on the patient's gradual but noticeable clinical improvement with the treatment, the dialysis catheter was retained. He was eventually discharged home with intraperitoneal antibiotics vancomycin and cefepime for a total duration of three weeks, according to the ISPD guidelines.

## Discussion

Peritoneal dialysis (PD) is frequently associated with the dangerous complication of peritonitis. For more than 6% of PD patients, PD-related peritonitis is the primary contributing cause of mortality [1].

According to a large-scale observational study conducted in the United States, approximately 62% of peritonitis cases are caused by gram-positive bacteria, of which 31% are coagulase-negative staphylococcus, 20.5% are caused by gram-negative bacteria, evenly split between Escherichia coli, Klebsiella species, and Pseudomonas aeruginosa. Fungi cause 3.92% of PDAP and 15.9% are reported as culture-negative peritonitis and probably higher in few other cases [5-7]. In this article, we describe a rare instance of PD-related peritonitis brought on by Neisseria sicca subspecies of Neisseria. The Neisseria genus includes multiple less well-studied species, commonly referred to as "commensals", and Neisseria sicca is one among them. N. sicca is a fastidious gram-negative diplococci that is commonly known to colonize the human nasopharynx and immunocompromised hosts are known to be vulnerable to this opportunistic organism [8,9].

One can broadly categorize peritoneal dialysis-associated peritonitis based on the cause, whether it is due to the translocation of bacteria from the gut into the peritoneal fluid, direct extension from the catheter, or hematogenous seeding due to bacteremia from another cause. Peritoneal dialysis-associated peritonitis and catheter-related infections, like exit-site and tunnel infections, are known to be associated [3,10,11]. Catheter-related peritonitis can be diagnosed when it occurs concomitantly or within three months with a documented exit-site and/or tunnel infection [3]. In the case of recent antibiotic exposure, catheter-related peritonitis can result in one site (e.g., exit-site or PD effluent) being culture-negative [3]. Wet contamination is the term used to describe contamination with an open system that occurs when the catheter administration set is kept open for a long time or when dialysis fluid is infused after contamination. Therefore, information about handling of the catheter holds importance to identify tunnel contamination, which applies in this case as the patient reported leaving the catheter open a few days before this episode, which may have led to the current infection. The most likely cause of N. sicca peritonitis in this patient is the contamination of the catheter by the hands due to a lack of sterile technique [3].

Neisseria sicca-associated peritonitis is uncommon. One case in the literature reported a Staphylococcus aureus PDAP episode followed by N. sicca. In that case, empiric intraperitoneal (IP) vancomycin was initiated for the first episode and then empiric IP vancomycin was followed by IP ceftazidime for the second episode once the organism was identified. Symptoms resolved 48 hours after ceftazidime initiation with normal dialysis effluent analysis and cultures seven days later. Another case reported an 83-year-old male with similar complaints who was started on empiric IP vancomycin with brief improvement followed by return of symptoms. Ceftazidime 500 milligrams/liter intravenous every day was later added. Subsequently, the culture grew Neisseria sicca/subflava, ceftazidime was discontinued, and ceftriaxone 750 milligrams intravenous every 12 hours was initiated. The patient had a complete resolution of peritonitis with normalization of fluid analysis and negative cultures on day 10 [9,11].

**Figure 3:**
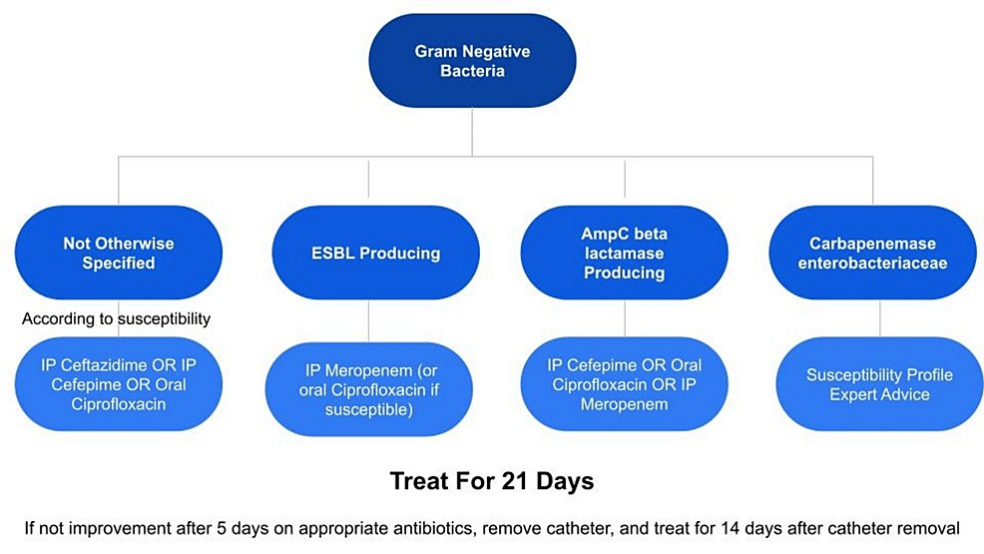
Flow chart depicting the antibiotic duration for managing gram-negative bacteria. Adapted from Ref. [3].

Identifying the organism causing PDAP and determining its subsequent antibiotic sensitivity, if possible, aids in choosing the appropriate antibiotic as well as potentially revealing the source of the peritonitis. Unfortunately, standard microbiology laboratories do not perform susceptibilities on N. sicca isolates. There are no interpretive minimum inhibitory concentration breakpoints. No specific drug of choice is recommended for N. sicca in the guidelines or in the literature. It appears there are rising rates of resistance in commensal Neisseria species to penicillins, macrolides, fluoroquinolones, and possibly third-generation cephalosporins making management difficult [12]. This patient was treated based on guideline recommendations for gram-negative bacteria for the required duration, as shown in Figure 3 below.

In the five months after the patient was treated for this episode, there have been no visits to the clinic or hospital with peritonitis or symptoms concerning for recurrence. The patient is undergoing a kidney transplant evaluation.

## Conclusions

Peritonitis is a significant and potentially life-threatening complication in patients on peritoneal dialysis. Prompt recognition of the aetiology is vital for proper management and favorable outcomes. PDAP is diagnosed based on ISPD guidelines by the presence of two out of three criteria, the presence of clinical features consistent with peritonitis such as abdominal pain and/or cloudy dialysis effluent, effluent with WBC count greater than 100/µL or > 0.1 × 10^9^/L with greater than 50% PMN, and a positive dialysis effluent culture. Although this is not the first report of Neisseria sicca peritonitis in an adult patient undergoing continuous ambulatory peritoneal dialysis, it is an uncommon pathogen and treatment is not standardized. It is imperative to use culture data to guide PDAP treatment whenever possible and to consider uncommon pathogens if the patient fails to respond. Identifying the organism causing PDAP and determining its subsequent antibiotic sensitivity, if possible, aids in choosing the appropriate antibiotic as well as potentially revealing the source of the peritonitis.
